# Dopamine D2L Receptor Deficiency Alters Neuronal Excitability and Spine Formation in Mouse Striatum

**DOI:** 10.3390/biomedicines10010101

**Published:** 2022-01-04

**Authors:** Gubbi Govindaiah, Rong-Jian Liu, Yanyan Wang

**Affiliations:** 1 Department of Anatomical Sciences and Neurobiology, School of Medicine, University of Louisville, Louisville, KY 40292, USA; iahgovinda494@gmail.com; 2 Department of Psychiatry, Yale University School of Medicine and Connecticut Mental Health Center, New Haven, CT 06508, USA; rong-jian.liu@yale.edu; 3 Department of Pharmaceutical Sciences & Health Outcomes, Ben Maytee Fisch College of Pharmacy, University of Texas at Tyler, Tyler, TX 75799, USA; 4 Department of Medical Information Science, UI College of Medicine, University of Illinois at Urbana-Champaign, Urbana, IL 61801, USA

**Keywords:** dopamine D2 receptor, dopamine D2L knockout mice, excitability of neurons, dendritic spine, cholinergic interneuron, medium spiny projection neuron

## Abstract

The striatum contains several types of neurons including medium spiny projection neurons (MSNs), cholinergic interneurons (ChIs), and fast-spiking interneurons (FSIs). Modulating the activity of these neurons by the dopamine D2 receptor (D2R) can greatly impact motor control and movement disorders. D2R exists in two isoforms: D2L and D2S. Here, we assessed whether alterations in the D2L and D2S expression levels affect neuronal excitability and synaptic function in striatal neurons. We observed that quinpirole inhibited the firing rate of all three types of striatal neurons in wild-type (WT) mice. However, in D2L knockout (KO) mice, quinpirole enhanced the excitability of ChIs, lost influence on spike firing of MSNs, and remained inhibitory effect on spike firing of FSIs. Additionally, we showed mIPSC frequency (but not mIPSC amplitude) was reduced in ChIs from D2L KO mice compared with WT mice, suggesting spontaneous GABA release is reduced at GABAergic terminals onto ChIs in D2L KO mice. Furthermore, we found D2L deficiency resulted in reduced dendritic spine density in ChIs, suggesting D2L activation plays a role in the formation/maintenance of dendritic spines of ChIs. These findings suggest new molecular and cellular mechanisms for causing ChIs abnormality seen in Parkinson’s disease or drug-induced dyskinesias.

## 1. Introduction

The striatum, a main input nucleus of the basal ganglia, plays an important role in motor control, reward processing, and habit formation [1,2] and is involved in the pathophysiology of movement disorders, such as Parkinson’s disease (PD) and dyskinesias [3,4]. The physiological properties and function of neurons in the striatum are heterogeneous. Medium-sized spiny neurons (MSNs) constitute approximately 95% of the total neuronal population in rodent striatum [5]. MSNs are GABAergic projection neurons. They send efferents to other basal ganglia nuclei through two main pathways: a direct pathway mediated by the dopamine D1 receptor (D1R), and an indirect pathway mediated by the dopamine D2 receptor (D2R) [6,7]. The direct pathway sends projections from the striatum directly to the substantia nigra pars reticulata (SNr). The indirect pathway sends projections from the striatum to the globus pallidus that then sends projections to the subthalamic nucleus and the output signals from the subthalamic nucleus then reach SNr. The regulation of the striatal microcircuitry can have a great influence on the striatal outputs from the direct and indirect pathways [8,9]. An adequate balance between these two output pathways is crucial for motor control and various cognitive functions.

Although the relatively large-sized cholinergic interneurons (ChIs) represent a small percent (~2%) of the striatal neuronal population in rodents, they can exert a powerful influence on the activity of MSNs because their dense and widespread local axon collateral network primarily targets both direct pathway MSNs (dMSNs expressing D1R) and indirect pathway MSNs (iMSNs expressing D2R) [10,11,12]. ChIs can also influence the activity of fast-spiking interneurons (FSIs) through their local innervation. FSIs (~1%) can modulate the activity of MSNs via their local GABAergic innervation [13,14]. All three types of neurons are key components of the striatal microcircuit. These neurons can be distinguished by their morphological and electrophysiological characteristics [15].

Individual neurons can act as coincidence detectors that monitor the fluctuation of synaptic potentials and fire action potentials (spikes) when synaptic excitation brings the membrane potential to the spike threshold. The rate of spike firing is influenced by the fluctuation of intrinsic excitability, a form of neuronal plasticity. Increased intrinsic excitability would operatively lower the threshold of generating action potential and thereby enhance the neuron’s responsiveness to subsequent synaptic excitation. The overall synaptic input activity results from the integration of excitatory and inhibitory synaptic inputs. Evidence suggests that alterations in neuronal excitability may be a cellular correlate of behavioral events [16] or a cellular mechanism underlying pathological conditions [17].

D2R is expressed on ChIs, a portion of MSNs, cortical glutamatergic afferents, and GABAergic terminals onto ChIs in the striatum [18,19,20,21]. Electrophysiological studies have revealed dopamine (DA) and the preferential D2R agonist quinpirole depress evoked GABA-mediated inhibitory postsynaptic currents (IPSCs) recorded in ChIs or some MSNs in rat or mouse striatum, and this effect is mediated by D2R via a presynaptic mechanism [22,23,24,25].

Alternative splicing generates two D2R isoforms, D2L (long form) and D2S (short form) [26]. To understand the specific function of D2L and D2S in the mammalian central nervous system (CNS), we generated dopamine D2L knockout (D2L KO) mice. Using various approaches, we demonstrated that D2L KO mice still express a functional D2S isoform on the cell surface [27,28]. Previously we reported that D2L KO mice displayed a number of robust behavioral phenotypes distinct from WT mice. For example, we showed that D2L may play a greater role than D2S in motor activity, opiate addiction, and associative and reversal learning [27,29,30]. On the other hand, D2S may contribute more than D2L to amphetamine-induced stereotyped behaviors and prepulse inhibition and L-dopa-induced abnormal involuntary movements [28,31,32]. These indicate that D2L and D2S indeed have differential functions in the CNS [27,33]. However, the impact of the altered expression level of D2L and D2S on the components of the striatal microcircuit at the cellular and synaptic level remains unclear.

The purposes of this study were to examine whether alterations in D2L and D2S expression levels would affect neuronal excitability, synaptic transmission, and spine formation in striatal neurons, using whole-cell recordings and two-photon imaging. This study will reveal how D2L deficiency or altered expression ratio of D2S to D2L may influence neuronal plasticity and synaptic modulation in the striatum. Such knowledge will provide insights into potential molecular and cellular mechanisms underlying behavioral alterations observed in D2L KO mice and enhance our understanding of the role of individual D2R isoforms in physiological and pathophysiological conditions.

## 2. Materials and Methods

### 2.1. Animals

D2L KO mice were generated as described previously [27]. D2L KO mice were backcrossed to the *C57BL/6* strain for eight generations to produce an incipient congenic B6 line. Wild-type (WT) mice were produced from WT littermates by mating D2L heterozygotes. Mice were maintained on a 12-h light/dark cycle (7 a.m./7 p.m.) in a temperature- and humidity-controlled room and had access to food and water *ad libitum*. All experimental procedures were approved by the Institutional Animal Care and Use Committee and conducted in accordance with the National Institute of Health Guide for the Care and Use of Laboratory Animals.

### 2.2. Brain Slice Preparation

Brain slices containing the dorsal striatum (caudate-putamen) were prepared from WT and D2L KO mice (age: 14–24 days) [15,34]. Animals were deeply anesthetized with sodium pentobarbital (50 mg/kg) and decapitated, and brains were submerged in cold (~4 °C), oxygenated (95% O_2_/5% CO_2_) slicing solution containing (in mM): 2.5 KCl, 26 NaHCO_3_, 1.25 NaH_2_PO_4_, 10 MgCl_2,_ 2 CaCl_2,_ 234.0 sucrose, and 11.0 glucose. Coronal slices (250–300 µM thickness) at the level of the striatum were cut using a vibrating tissue slicer (Leica 1000S) and incubated for at least one hour prior to recording. A recovery period of 1–2 hr was allowed before the commencement of recording. Individual slices were then transferred to a recording chamber and were superfused with a physiological solution containing (in mM): 126.0 NaCl, 26 NaHCO_3_, 2.5 KCl, 1.25 NaH_2_PO_4_, 2.0 MgCl_2_, 2.0 CaCl_2_, and 10.0 glucose. The solution was oxygenated (95% O_2_/5% CO_2_) and maintained at 32 °C.

### 2.3. Whole-Cell Recordings and Data Analysis

Whole-cell recordings were performed on neurons in the dorsal striatum in oxygenated physiological solution at 32 °C [15]. Recordings were obtained with the visual aid of a microscope (Axioskop 2FS, Carl Zeiss, Inc., Thornwood, NY, USA) equipped with differential interference contrast optics. Recording pipettes were pulled from borosilicate glass capillaries and had tip resistances of 3–5 MΩ when filled with an internal solution. Two types of internal solutions were used. To examine neuronal excitability and miniature excitatory postsynaptic currents (mEPSCs), the potassium-based internal solution was used that contained (in mM): 117.0 K-gluconate, 13.0 KCl, 1.0 MgCl_2_, 0.07 CaCl_2_, 0.1 EGTA, 10.0 HEPES, 2.0 Na_2_-ATP and 0.4 Na-GTP and 0.3% biocytin. To record miniature inhibitory postsynaptic currents (mIPSCs), the cesium-based internal solution was used for voltage-clamp recordings that contained (in mM): 117.0 Cs-gluconate, 13.0 CsCl, 1.0 MgCl_2_, 0.07 CaCl_2_, 0.1 EGTA, 10.0 HEPES, 2.0 Na_2_-ATP and 0.4 Na-GTP and 0.3% biocytin. When the Cs-based solution was used, the electrode tips were first filled with the K-based internal solution. The pH and osmolarity of the internal solution were adjusted to 7.3 and 290 mOsm, respectively.

Neuronal types were distinguished based on morphological characteristics and electrophysiological properties as described previously [15]. After forming the whole cell configuration, the recording was allowed to stabilize for at least 5 min prior to data acquisition. Action potentials (spike firing) were induced by injection of depolarizing currents in whole-cell current-clamp mode. To evaluate the effects of quinpirole on spike firing in striatal neurons, depolarizing current steps (50–200 pA, 600 ms) were applied through the patch pipette in current-clamp mode. mIPSCs and mEPSCs were recorded in ChIs in the presence of tetrodotoxin (TTX; 0.5 µM). mIPSCs were recorded using a Cs-based internal solution at a holding potential of 0 mV to optimize IPSC recordings. mEPSCs were recorded using a K-based internal solution at a holding potential of −70 mV.

Recordings were performed using a Multiclamp 700B amplifier (Molecular Devices, Sunnyvale, CA, USA), sampled at 2.5–5 kHz, low-pass filtered at 10 kHz using Digidata 1440 digitizer. Electrophysiological data were stored on a computer for subsequent analyses using pClamp software. Bridge balance (current-clamp recordings) and series resistance (voltage-clamp recordings) were continuously monitored and properly compensated [35]. Data were not included in the analyses if the access resistance was changed by >15% during the recordings.

The frequency and amplitude of mIPSCs or mEPSCs were detected and analyzed offline using MiniAnalysis software (Synaptosoft, Leonia, NJ, USA) as described previously [36]. The threshold for mIPSC detection was set at 10 pA and the automatic detection was verified post hoc by visual inspection.

### 2.4. Imaging and Data Analysis

Pipettes (3–5 MΩ) were first tip-filled with regular K-based internal solution before backfilling with the internal solution containing 0.3% neurobiotin (Vector Laboratories, Burlingame, CA, , USA) to avoid ejecting excess dye into the extracellular space of striatal slices. Cells were held around their resting membrane potential in whole-cell current-clamp mode. After the completion of ejecting neurobiotin solution into cells, slices were transferred to 4% paraformaldehyde in 0.1 M phosphate buffer and stored overnight at 4 °C. Slices were then processed with streptavidin conjugated to Alexa 594 (1:1000; Invitrogen, Waltham, MA, USA) for neurobiotin visualization as previously described [37].

Imaging and analysis were performed according to the methods described previously [38]. Briefly, labeled neurons within the dorsal striatum were imaged with a two-photon laser scanning system. This consisted of a Ti:sapphire laser (Mai Tai; Spectra Physics, Santa Clara, CA, USA) tuned to wavelength 810 nm and a direct detection Bio-Rad Radiance 2100 MP laser scanner (Zeiss Microimaging, White Plains, NY, USA) mounted on an Olympus BX50WI microscope with ×40 (0.8 N.A.) or ×60 (0.9 N.A.) water-immersion objectives (Olympus, Bethlehem, PA, USA).

For spine density analyses, Z-stacks consisting of 1–10 scans at high zoom (68 × 68 μm; ×60 lens) at 1-μm steps in the *z*-axis were collected from the basal tuft arborization. Spine density was analyzed in the X-Y plane of projection images by using Laser Sharp 2000 software (Bio-Rad, Hercules, CA, USA). Spine density was sampled along dendritic branches of MSNs or ChIs. For each cell, 4–5 dendritic branches were examined.

### 2.5. Drugs

Concentrated stock solutions of pharmacological agents were prepared and stored as recommended by the manufacturer. Stock solutions were diluted in ACSF to final concentration just before use. Agonist was applied via a short bolus into the input line of the recording chamber using a syringe pump. The antagonist was bath applied for at least 5 min before subsequent experimental tests. Quinpirole and SR95531 were purchased from Sigma-Aldrich. All compounds were purchased from either Tocris Cookson (Ellisville, MO, USA) or Sigma-Aldrich (St. Louis, MO, USA).

### 2.6. Analysis and Statistics

Spike firing frequency was analyzed using Clampfit 9 software (pClamp9, Molecular Devices, Sunnyvale, CA, USA). For quantification of mIPSCs or mEPSCs, the average frequency of mIPSCs or mEPSCs was calculated from 60-s time windows. Statistical analyses were performed using either OriginLab Origin 8.5 or GraphPad Prism 8.0. Data from whole-cell recordings were compared between groups by using Student’s *t*-test or Kolmogorov–Smirnov test. Changes in spine density were compared between groups by using Student’s *t*-test. Data are presented as mean ± standard error of mean (SEM). A *p*-value < 0.05 is considered statistically significant.

## 3. Results

### 3.1. D2L Deficiency Produced Differential Effects on Neuronal Excitability in Three Types of Striatal Neurons

**Figure 1:**
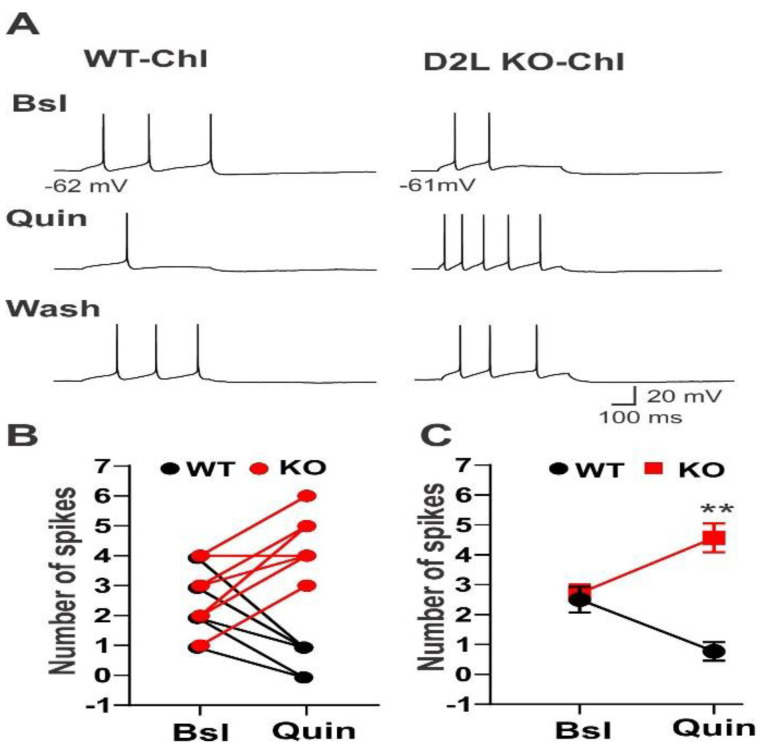
Quinpirole increased the excitability of ChIs in D2L KO mice. (**A**) Baseline (Bsl) spikes were induced by injecting depolarizing currents in ChIs from WT and D2L KO mice (top traces). Quinpirole (Quin) significantly decreased the firing rate in ChIs from WT mice (left middle trace), but significantly increased the firing rate in ChIs from D2L KO mice (right middle trace). Wash: spikes were recorded after washing off quin (bottom traces). (**B**) Summary of quinpirole effects on spike firing of ChIs from WT mice (filled black circles, *n* = 6) and from D2L KO mice (filled red circles, *n* = 7). The spike numbers of individual cells were plotted versus Bsl (without quin) or in the presence of Quin. (**C**) The mean spike numbers of cells were plotted versus Bsl or Quin. ** *p* < 0.003 (WT); ** *p* < 0.006 (D2L KO; Student’s *t*-test). Note that some lines are overlapped in Figure 1B, Figure 2B and Figure 3B.

To assess if the deletion of D2L would alter the excitability of striatal neurons, we examined the effect of quinpirole, a preferential D2R agonist, on three types of striatal neurons: MSNs, ChIs, and FSIs. We first examined the excitability of ChIs in the absence or presence of quinpirole. Quinpirole (3 µM) significantly decreased the firing rate in ChIs from WT mice (Figure 1A left traces). The mean firing rate was decreased to 0.76 ± 0.31 Hz in the present of quinpirole from the baseline frequency of 2.5 ± 0.42 Hz (*n* = 6; *p* < 0.003; Student’s *t*-test) (Figure 1B,C). In contrast, quinpirole significantly increased the firing rate to 4.57 ± 0.48 Hz from the baseline frequency of 2.71 ± 0.32 Hz in ChIs from KO mice (*n* = 7; *p* < 0.006) (Figure 1A–C).

**Figure 2:**
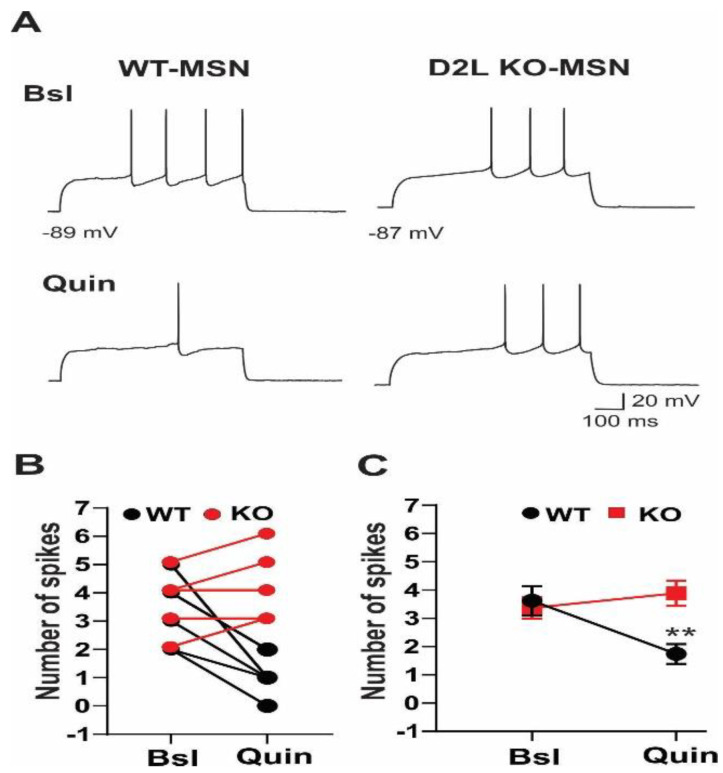
Effect of quinpirole on spike firing of MSNs was absent in D2L KO mice. (**A**) Baseline (Bsl) spikes were induced by injecting depolarizing currents in MSNs from WT and D2L KO mice (top traces). Quinpirole (Quin) significantly decreased the firing rate in MSNs from WT mice (left middle trace), but it had no effect on the firing rate in MSNs from D2L KO mice (right middle trace). (**B**) Summary of quinpirole effects on spike firing of MSNs from WT mice (filled black circles, *n* = 7) and from D2L KO mice (filled red circles, *n* = 8). The spike numbers of individual cells were plotted versus Bsl (without quin) or in the presence of Quin. (**C**) The mean spike numbers were plotted versus Bsl or Quin. ** *p* < 0.002 (WT); *p* > 0.3 (D2L KO).

We next tested the effect of quinpirole on the excitability of MSNs. Quinpirole significantly decreased the firing rate in MSNs from WT mice (Figure 2A left traces). The mean firing rate was decreased to 1.75 ± 0.35 Hz in the presence of quinpirole from the baseline frequency of 3.62 ± 0.41 Hz in MSNs from WT mice (*n* = 7; *p* < 0.002) (Figure 2B,C). However, quinpirole had no significant effect on the firing rate in MSNs from D2L KO mice (Bsl: 3.37 ± 0.38 Hz, *n* = 8; Quin: 3.87 ± 0.39 Hz, *n* = 8; *p* > 0.3) (Figure 2A–C).

**Figure 3:**
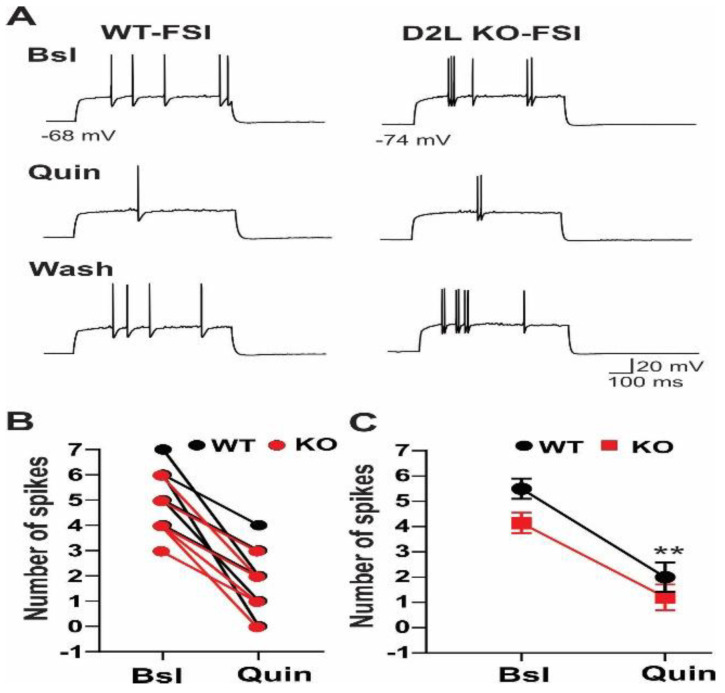
Effect of quinpirole on suppressing spike firing of FSIs was not altered in D2L KO mice. (**A**) Baseline (Bsl) spikes were induced by injecting depolarizing currents in MSNs from WT and D2L KO mice (top traces). The inhibitory effect of quinpirole (Quin) on the firing rate of FSIs was similar in WT (left middle trace) and D2L KO mice (right middle trace). Wash: spikes were recorded after washing off quin (bottom traces). (**B**) Summary of quinpirole effects on spike firing of FSIs from WT mice (filled black circles, *n* = 6) and from D2L KO mice (filled red circles, *n* = 8). The spike numbers of individual cells were plotted versus Bsl (without quin) or in the presence of Quin. (**C**) The mean spike numbers were plotted versus Bsl or Quin. ** *p* < 0.001 (WT); ** *p* < 0.001 (D2L KO).

Furthermore, quinpirole suppressed the spike firing in FSIs in both WT and D2L KO mice (WT: Bsl: 5.52 ± 0.42 Hz, Quin: 2.00 ± 0.57 Hz, *n* = 6; *p* < 0.001; D2L KO: Bsl: 4.14 ± 0.40 Hz, Quin: 1.14 ± 0.41 Hz, *n* = 8; *p* < 0.001) (Figure 3A–C). There was no significant difference in quinpirole-produced suppression of firing rate between WT FSIs and KO FSIs.

### 3.2. D2L Deficiency Resulted in a Decrease in mIPSC Frequency in Cholinergic Interneurons

**Figure 4:**
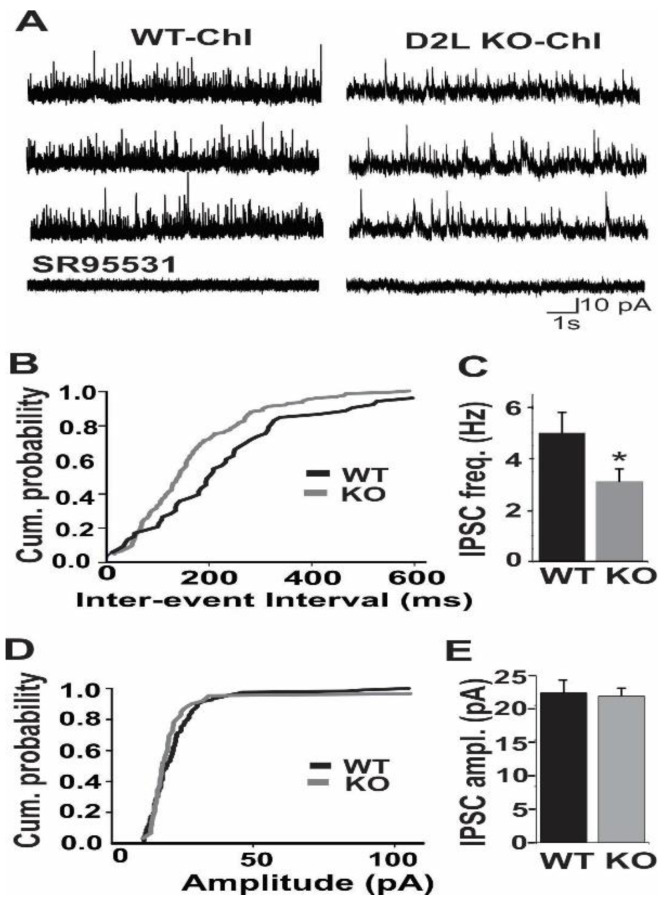
The frequency of mIPSCs in ChIs was significantly reduced in D2L KO mice. (**A**) Representative current traces recorded from ChIs of WT and D2L KO mice. mIPSCs were eleminated by SR95531, a GABA receptor antagonist. Cumulative probability plot (**B**) and summarized bar graph (**C**) reveal a significant reduction in mIPSC frequency in D2L KO mice (*n* = 8, * *p* < 0.01), compared with WT mice (*n* = 11). Cumulative probability plot (**D**) and summarized bar graph (**E**) reveal there is no significant difference in mIPSC amplitude between WT and D2L KO mice. WT (Black lines or bars) and D2L KO (Grey lines or bars).

To determine if D2L deficiency altered the synaptic activity of ChIs, we examined the frequency and amplitude of mIPSCs and mEPSCs in ChIs in the striatum. mIPSCs were recorded in ChIs using cesium-gluconate based internal solution at a holding potential of 0 mV. We found that the frequency of mIPSCs was significantly lower in D2L KO mice (2.9 ± 0.5 Hz, *n* = 8) than in WT mice (5.1 ± 0.6 Hz, *n* = 11) (*p* < 0.01; Kolmogorov–Smirnov test) (Figure 4A–C). On the other hand, there was no significant change in the amplitude of mIPSCs between D2L KO and WT mice (KO: 21.8 ± 1.6 pA; *n* = 8; WT: 22.4 ± 1.9 pA, *n* = 11; *p* > 0.08) (Figure 4A,D,E). These results indicated that spontaneous GABAergic inhibitory transmission was significantly reduced in ChIs in D2L KO mice.

**Figure 5:**
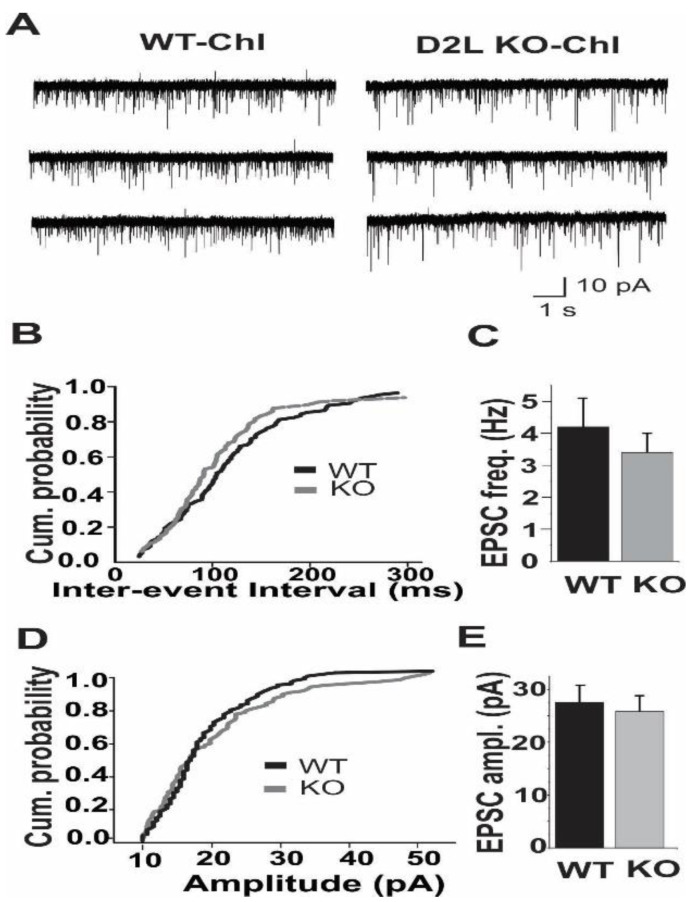
The frequency and amplitude of mEPSCs in ChIs were similar in WT and D2L KO mice. (**A**) Representative current traces recorded from ChIs of WT and D2L KO mice. Cumulative probability plot (**B**) and summarized bar graph (**C**) reveal there is no significant difference in mEPSC frequency between WT (*n* = 6) and D2L KO mice (*n* = 5) (*p* > 0.08). Cumulative probability plot (**D**) and summarized bar graph (**E**) reveal there is no significant difference in mEPSC amplitude between WT and D2L KO mice (*p* > 0.9). WT (Black lines or bars) and D2L KO (Grey lines or bars).

We then examined mEPSCs in ChIs in WT and D2L KO mice using K-gluconate based internal solution at a holding potential of −70 mV. We observed no significant changes in mEPSC frequency between WT and D2L KO mice (WT: 4.2 ± 0.9 Hz, *n* = 6; KO: 3.4 ± 0.7 Hz, *n* = 5, *p* > 0.08) (Figure 5A–C) or in mEPSC amplitude (WT: 27.6 ± 3.2 pA, *n* = 6; KO: 25.8 ± 3.5, *n* = 5, *p* > 0.09) (Figure 5A,D,E). These results indicated that spontaneous glutamatergic transmission in ChIs was not significantly altered.

### 3.3. D2L Deficiency Caused a Reduction in Dendritic Spine Density in Cholinergic Interneurons

**Figure 6:**
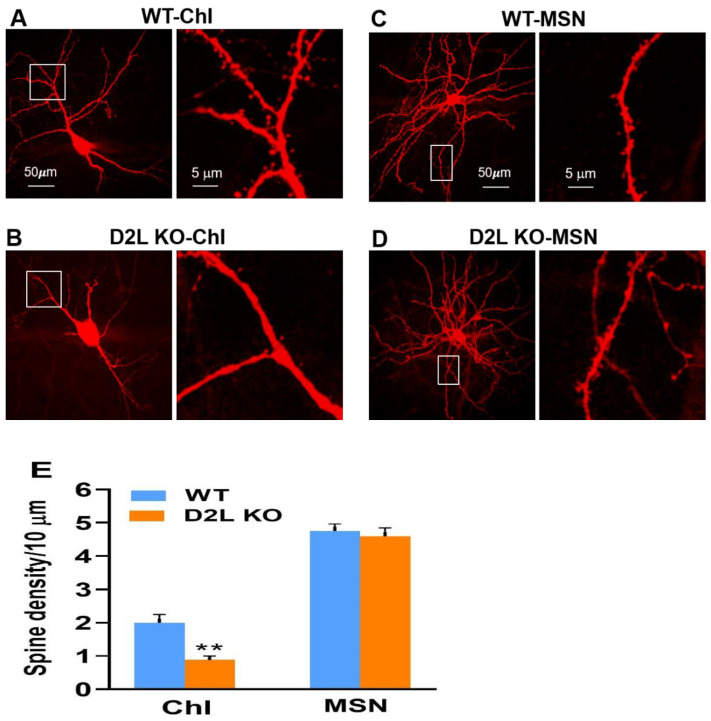
Dendritic spine density was reduced in striatal ChIs in D2L KO mice. (**A**) Two-photon images were taken from a representative ChI of WT mice. (**B**) two-photon images were taken from a representative ChI of D2L KO mice. Images shown on the right side (taken from white boxes in the left side images) are at a higher zoom level. (**C**) Two-photon images were taken from an MSN of WT mice. (**D**) two photon images were taken from an MSN of D2L KO mice. (**E**) Bar graph showing the mean (±SEM) of dendritic spine density in ChIs and MSNs from WT and D2L KO mice. There is a significant decrease in dendritic spine density of ChIs from D2L KO mice compared to those from WT mice (** *p* < 0.001). WT (Blue bars) and D2L KO (Orange bars).

Using two-photon imaging, we found that the deletion of D2L caused a significant decrease in the density of dendritic spines in ChIs from D2L KO mice compared with ones from WT mice (Figure 6A,B,E) (*p* < 0.001; *n* = 4 cells for WT; *n* = 3 cells for D2L KO; 4–5 dendritic branches were examined per cell). On the other hand, there was no significant change in the density of dendritic spines between WT MSNs and KO MSNs (Figure 6C, D, E) (*n* = 2 cells for WT and *n* = 2 cells for D2L KO). As summarized in Figure 6E, dendritic spine density was reduced by over 50% in ChIs from D2L KO mice, compared with that of WT mice.

Using cells filled with neurobiotin marker with streptavidin/Alexa 594 amplification, we found that ChIs from WT mice had spines on dendrites with the density about 2 spines per 10 µm of dendrite, mostly within about 200 µm from the soma (Figure 6A,E). The dendritic spine density of MSNs was about 4.75 spines per 10 µm of dendrite (Figure 6E).

## 4. Discussion

In the present study, we observed that in WT mice, the preferential D2R agonist quinpirole suppressed the firing rate in all three types of neurons in the striatum—ChIs, MSNs, and FSIs. However, D2L deficiency or increased ratio of D2S to D2L altered the effect of quinpirole on neuronal excitability depending on cell types. Our results showed that D2L deletion or increased ratio of D2S to D2L changed the quinpirole effect on ChIs from inhibition to excitation, thereby increasing the excitability of ChIs in D2L KO mice. On the other hand, the effect of quinpirole on spike firing of MSNs was absent in D2L KO mice. Moreover, the inhibitory effect of quinpirole on spike firing of FSIs remained unaltered in D2L KO mice.

In addition, D2L deficiency resulted in a decrease in the frequency of mIPSCs, but not the amplitude of mIPSCs, in ChIs from D2L KO mice compared to ones from WT mice. The frequency and amplitude of mEPSCs in ChIs remained similar in WT and D2L KO mice. The miniature inhibitory synaptic currents reflect quantal GABA release by spontaneous exocytosis [39]. The decreased frequency (but not amplitude) of mIPSCs suggests that spontaneous GABA release is reduced at the GABAergic terminals onto ChIs in D2L KO mice. It has been shown that receptors located at the presynaptic sites can modulate spontaneous neurotransmitter release [40]. There is a possibility that D2S may have a greater inhibitory effect than D2L on spontaneous GABA release. Decreased GABA release would result in the reduced inhibitory postsynaptic effect of GABA on ChIs. This may account, at least partially, for the enhanced excitability of ChIs in D2L KO mice.

Furthermore, using two-photon imaging, we found that D2L deficiency caused a reduction in the density of dendritic spines in ChIs. There is a possibility that the activation of D2L plays a significant role in the formation or maintenance of dendritic spines of ChIs. DA might have some trophic effects on the spine formation and/or maintenance of striatal ChIs through the activation of D2L. The loss of dendritic spines of ChIs may also contribute to the reduced GABAergic inhibitory inputs or influence on ChIs.

ChIs in the striatum are often referred to as large, aspiny cholinergic interneurons in the literature. Interestingly, in the present study, we were able to identify spines on the dendrites of ChIs, although the density is much lower than that of MSNs. This is likely because we filled cells with a neurobiotin marker that reliably filled every spine and the imaging was amplified with streptavidin/Alexa 594.

**Figure 7:**
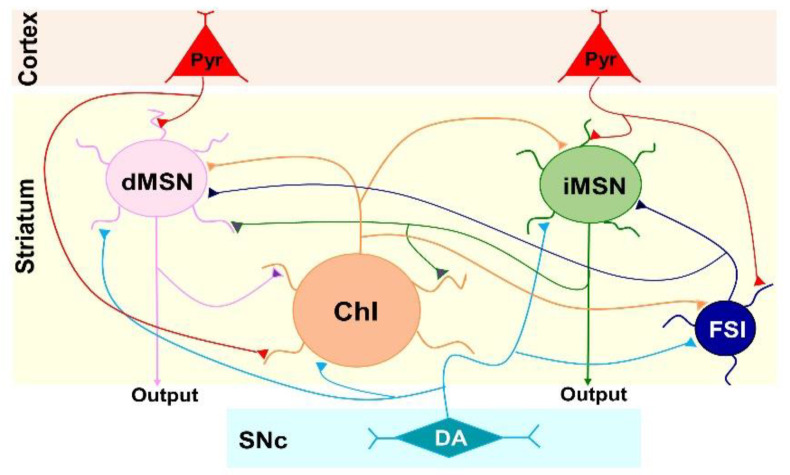
Schematic illustration of the microcircuit of the striatum. dMSN, direct pathway MSN; iMSN, indirect pathway MSN; ChI, cholinergic interneuron; FSI, fast-spiking interneuron; Pry, pyramidal neuron in the cortex; DA, dopaminergic neuron in the substantia nigra compacta (SNc). The axonal inputs are representatives; some inputs could target to both somata and dendrites.

MSNs, ChIs, and FSIs together account for approximately 98% of neurons in the striatum. All three types of neurons can reciprocally modulate each other’s activity via local synaptic connections and are key modules in the striatal microcircuit [17,41] (Figure 7). dMSNs and iMSNs can also influence each other’s activity through local axon collaterals [9]. All three types of striatal neurons receive excitatory glutamatergic inputs from the cortex and dopaminergic inputs from the substantia nigra compacta (SNc) [8]. The increased intrinsic excitability of ChIs showed in the present study would likely lead to an increase in the release of acetylcholine (ACh), which in turn activates muscarinic and nicotinic receptors located on various cells, glutamatergic afferents, or dopaminergic afferents in the striatum [42,43,44]. Consequently, this would cause a disruption in neural network dynamics within the striatum as well as an imbalance between dMSN- and iMSN- mediated outputs.

The striatum is involved in the pathophysiology of movement disorders, such as PD and drug-induced dyskinesias [3,4,45]. Previously we showed that D2L KO mice displayed enhanced abnormal involuntary movements (AIMs) in response to chronic treatment of L-dopa or quinpirole [32]. AIMs are used as an experimental model for investigating the mechanisms of drug-induced dyskinesia. In the present study, we found that the intrinsic excitability of ChIs was increased in D2L KO mice compared with WT mice. The increased excitability of ChIs in the striatum may provide a cellular mechanism underlying the enhanced L-dopa-induced AIMs in D2L KO mice reported in our previous study [32]. Earlier studies by other scientists have shown that increasing the firing rate of ChIs or activating ChIs by optogenetic stimulation in the striatum results in an enhancement in L-dopa-induced dyskinesia in mice [46,47]. Our studies support the findings of the earlier studies by others.

Furthermore, our findings shed light on a new molecular and cellular mechanism for causing the hyperactivity of ChIs. It is known that in PD, DA release is reduced in the striatum due to the degeneration of dopaminergic neurons in the SNc, and this results in an imbalance between dopaminergic and cholinergic activity in the striatum [44,48]. Our present results suggest additional factors contributing to the imbalance between dopaminergic and cholinergic activity in striatum-related pathological conditions. There is a possibility that decreased D2L activity may cause a retraction in dendritic spines in ChIs and a reduction in spontaneous GABAergic inputs/influence on ChIs, consequently leading to ChIs hyperactivity. We cannot completely exclude the possibility that D2S may have an excitatory effect on ChIs. Regardless, we showed that D2L deficiency or increased ratio of D2S to D2L not only affected the activity of iMSNs, but also had impacts on the activity of other modules in the striatal microcircuit, such as ChIs. Our previous and present studies together suggest alterations in D2L and/or D2S expression could lead to ChIs hyperactivity, which may contribute to the development and/or expression of dyskinesia induced by dopaminergic drugs.

In conclusion, our results showed that D2L deficiency or increased expression ratio of D2S to D2L could lead to an enhancement in the excitability of ChIs due, at least in part, to the decreased spontaneous GABA release and the loss of dendritic spines in ChIs. Our findings reveal new molecular and cellular mechanisms for what could result in the abnormalities of ChIs and have implications for further understanding the pathophysiology of striatum-related disorders, such as PD and/or dopaminergic drug-induced dyskinesias. A better understanding of individual D2R isoforms in neuronal excitability and synaptic modulation will help us gain novel insights into the regulation of the striatal microcircuit. This may facilitate designing improved therapeutic strategies or agents for the treatment of dopaminergic related CNS disorders.

## Data Availability

The data presented in this study are available on request from the corresponding author.
